# A dynamically loaded *ex vivo* model to study neocartilage and integration in human cartilage repair

**DOI:** 10.3389/fcell.2024.1449015

**Published:** 2024-09-30

**Authors:** Anna Trengove, Lilith M. Caballero Aguilar, Claudia Di Bella, Carmine Onofrillo, Serena Duchi, Andrea J. O’Connor

**Affiliations:** ^1^ BioFab3D@ACMD, St Vincent’s Hospital Melbourne, Fitzroy, VIC, Australia; ^2^ Department of Biomedical Engineering, The Graeme Clark Institute, The University of Melbourne, Parkville, VIC, Australia; ^3^ Department of Orthopaedics, St Vincent’s Hospital Melbourne, Fitzroy, VIC, Australia; ^4^ Department of Surgery, The University of Melbourne, Parkville, VIC, Australia

**Keywords:** cartilage, integration, bioreactor, bioadhesive, hydrogels, chondrogenesis, mechanical stimulation, dynamic loading

## Abstract

Articular cartilage injuries in the knee can lead to post-traumatic osteoarthritis if untreated, causing debilitating problems later in life. Standard surgical treatments fail to ensure long lasting repair of damaged cartilage, often resulting in fibrotic tissue. While there is a vast amount of research into cartilage regeneration, integrating engineered implants with cartilage remains a challenge. As cartilage is a load bearing tissue, it is imperative to evaluate tissue repair strategies and their ability to integrate under mechanical loading. This work established a dynamically loaded *ex vivo* model of cartilage repair using human cartilage explants. The model was used to assess the efficacy of a stem cell therapy delivered in a bioadhesive hydrogel comprised of photocrosslinkable gelatin methacryloyl (GelMA) and microbial transglutaminase to repair the model defect. Extensive neocartilage production and integration were observed via histology and immunohistochemistry after 28 days chondrogenic culture. Analysis of culture media allowed monitoring of glycosaminoglycan and type II collagen production over time. A mechanical assessment of integration via a push out test showed a 15-fold increase in push out strength over the culture duration. The model was successful in exhibiting robust chondrogenesis with transglutaminase or without, and under both culture conditions. The work also highlights several limitations of *ex vivo* models and challenges of working with bioreactors that must be overcome to increase their utility. This *ex vivo* model has the potential to delay the need for costly pre-clinical studies and provide a more nuanced assessment of cartilage repair strategies than is possible *in vivo*.

## 1 Introduction

Injury to the knee can create defects in articular cartilage, which are reported in approximately 60% of patients undergoing knee arthroscopy (Merkely et al., 2018). As cartilage has little to no ability to self-repair, these defects can progress to post traumatic osteoarthritis, with the end stage requiring a total joint replacement (Anderson et al., 2011). Existing surgical treatments such as microfracture or osteochondral grafts see improvement in clinical outcomes in the short term, but do not generally provide a long-lasting repair (Solheim et al., 2018; Epanomeritakis et al., 2022). A significant challenge in all cartilage repair treatments is host integration, though cell-based therapies do show promise (Khan et al., 2008; Huey et al., 2012; Epanomeritakis et al., 2022; Trengove et al., 2022).

Tissue engineering strategies have demonstrated generation of cartilage tissue *in vitro* and *in vivo*, via bench-based fabrication of scaffolds (which can then be implanted) or via *in situ* fabrication (Wu et al., 2018; Kwon et al., 2019; Wei et al., 2021). However, neocartilage generated with these approaches struggles to integrate with native cartilage *in vivo* and this remains an issue for clinical translation (Wilke et al., 2007; Di Bella et al., 2018; Kwon et al., 2019). The use of an adhesive may provide a solution to this problem, by securing an implant in the cartilage defect. Indeed, we previously demonstrated microbial transglutaminase (TG) significantly improved bioadhesion of gelatin methacryloyl (GelMA) hydrogel to human cartilage explants (Trengove et al., 2021). Transglutaminase is a relatively low-cost enzyme used as a food additive in many countries; shows evidence of biocompatibility *in vitro*; and is understood to support adhesion by catalysing the formation of covalent bonds between glutamine and lysine residues found in both GelMA and cartilage (Trengove et al., 2021).

The ability to withstand daily loading of the joint is a core function of articular cartilage, however the vast majority of *ex vivo* cartilage repair models are cultured under static conditions (Maher et al., 2010; Erickson et al., 2012; Athens et al., 2013; Broguiere et al., 2016; Kuang et al., 2019; Galarraga et al., 2021). Spinner flask bioreactors or bioreactors applying mechanical loads can create an environment that is more physiologically relevant, given the role of joint loading in maintaining healthy cartilage metabolism (Sermer et al., 2018; Vincent and Wann, 2019). Previous research in an *ex vivo* model of cartilage repair has found a complex relationship between cyclic loading and integration of a scaffold with cartilage, with loading having constructive or destructive effects depending on the magnitude of the load and the initial degree of integration (e.g., due to pre-culture) (Yodmuang et al., 2019). It is unclear if a bioadhesive can provide initial fixation to support improved integration and regeneration of a cartilage defect in an *ex vivo* model under loading. It should also be noted that many current *ex vivo* models of cartilage repair use animal tissue and inter-species differences in anatomy may diminish the relevance of findings from these studies (Trengove et al., 2024). Thus, a dynamically loaded model utilizing human cartilage explants could be a powerful tool to screen the efficacy of cartilage repair strategies during development, rather than the use of animal models, which are costly, raise ethical issues, and the results of which may not extrapolate well to humans (Xing et al., 2016; Trengove et al., 2024).

This work aims to establish a dynamically loaded *ex vivo* model utilizing human cartilage explants, and subsequently aims to assess the efficacy of a bioadhesive cell-laden hydrogel for regenerating cartilage. We hypothesised that the bioadhesive will help to stabilise the cell-laden hydrogel in a model cartilage defect, seeing improved integration under loading compared to the bioadhesive-free control. A model is used where a cartilage defect is created in cartilage discs to better study the effect of the adhesion of the hydrogel specifically to the human cartilage tissue via cyclic compression and mechanical analysis. Gelatin methacryloyl (GelMA) was used to encapsulate human adipose derived stem cells (hADSCs) in a human cartilage explant with transglutaminase added to the hydrogel for bioadhesion, building on work to investigate a therapy to repair chondral defects (Di Bella et al., 2018; Onofrillo et al., 2018; Trengove et al., 2021; Onofrillo et al., 2021). The team have previously performed *in vivo* rabbit (Onofrillo et al., 2021; Duchi et al., 2023) and sheep (Di Bella et al., 2018) animal studies and demonstrated cartilage repair in a full chondral defect model. We also hypothesized that the dynamic *ex vivo* model could provide information on neocartilage production through non-destructive media analyses. Cartilage regeneration and integration were evaluated following 28 days culture in chondrogenic media within a bioreactor applying cyclic compressive loading. Loading was intended to provide a physiologically relevant strain of the interface, akin to motion or partial weight bearing following surgery. These results bring to light the chondrogenic potential of this repair strategy, and the potential of this *ex vivo* model to assess cartilage tissue engineering strategies.

## 2 Materials and methods

### 2.1 Materials

Gelatin methacryloyl (GelMA) (TRICEP, Wollongong, NSW, Australia) was sterilised using ethylene oxide (EtO) gas in an Anprolene EtO steriliser (Andersen Sterilizers Inc., United Kingdom). Lithium phenyl-2,4,6-trimethylbenzoylphosphinate (LAP) (Tokyo Chemical Industries, Tokyo, Japan) was dissolved in phosphate buffered saline (PBS, Gibco, Thermo Fisher Scientific Inc., Waltham, MA, United States) (4% w/v) containing 100 U/mL penicillin and 100 μg/mL of streptomycin (pen-strep) (Gibco, Thermo Fisher Scientific Inc.). Stock solution of microbial transglutaminase (TG) (Moo Gloo TI Transglutaminase, Modernist Pantry, Portsmouth, New Hampshire) was prepared in PBS (10% w/v) and filter sterilized through a 33 mm diameter polyethersulfone syringe filter with 0.22 µm pore size (Millex-GP, Merck, Darmstadt, German). Transglutaminase specific enzyme activity was ∼100 U/g in the stock solution as measured by a colorimetric hydroxamate assay (Caramez Triches Damaso et al., 2013).

### 2.2 Stem cell isolation and culture

Human adipose derived stem cells (hADSCs) were isolated from infrapatellar fat pad obtained from consenting patients undergoing total knee replacement surgery with grade 3 osteoarthritis according to the Kellgren-Lawrence grading scale, a commonly used system for classifying the severity of osteoarthritis (OA) based on radiographic features, using a previously described protocol (Kellgren and Lawrence, 1957; Ye et al., 2014). The use of human samples and procedures in this study was approved by the Human Research Ethics Committee Research Governance Unit of St. Vincent’s Hospital, Melbourne, Australia [HREC/16/SVHM/186]. All experiments were performed in accordance with relevant guidelines and regulations. Isolated cells were expanded in growth media comprising low glucose DMEM (Sigma-Aldrich) supplemented with 10% FBS (Gibco), pen-strep, 2 mM L-glutamine (Gibco), 15 mM HEPES, 20 ng/mL epidermal growth factor (EGF) and 1 ng/mL fibroblast growth factor (FGF-2) (R&D Systems Inc., Minneapolis, MN, United States).

### 2.3 Preparation of cartilage explants for *ex vivo* model

**FIGURE 1:**
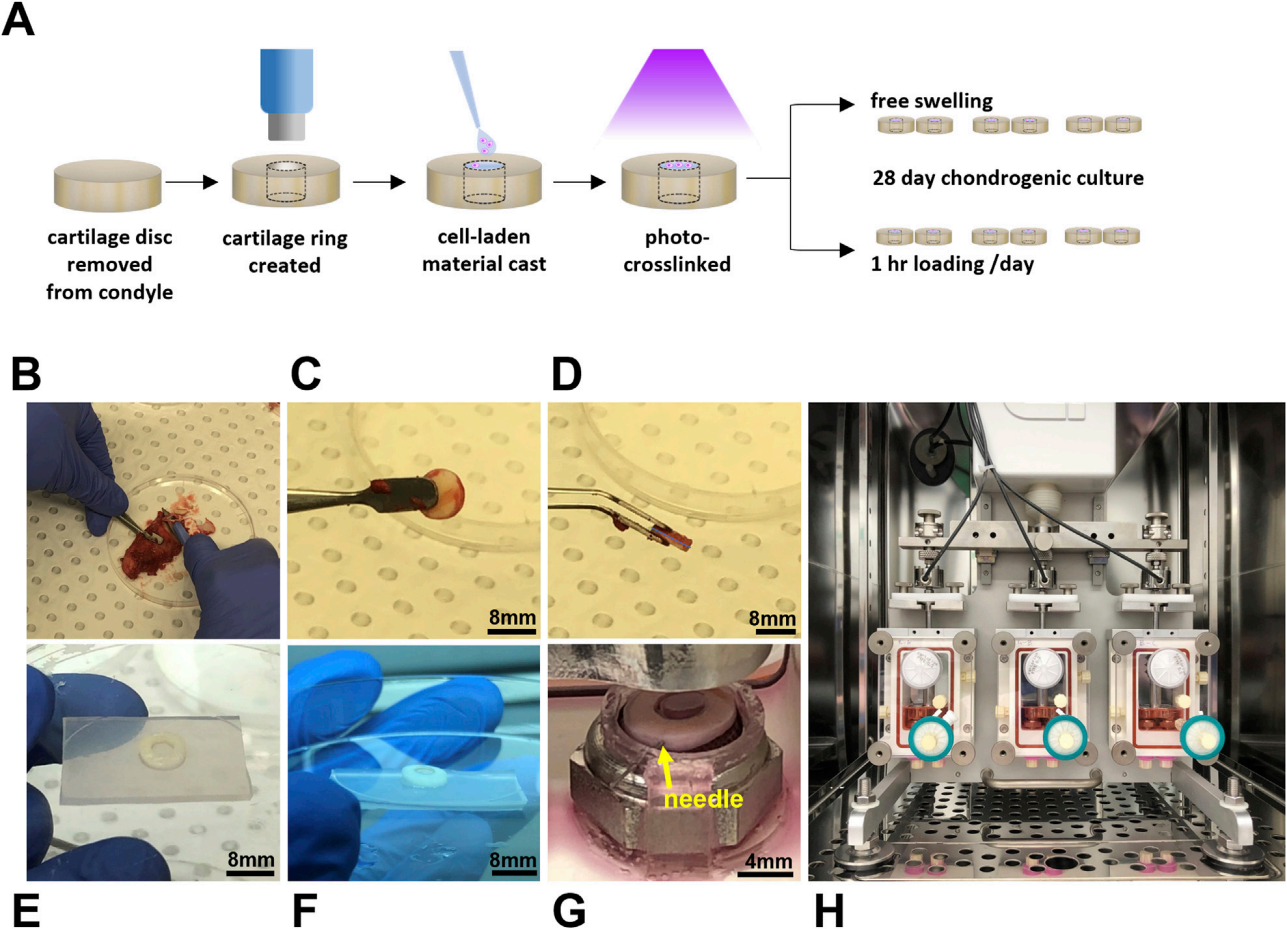
Dynamically loaded human *ex vivo* model of cartilage repair. Schematic illustrating experimental workflow **(A)** and images **(B–E)** illustrating the preparation of cartilage rings via the creation of a defect in a disc of human cartilage explanted following total knee replacement surgery. Cartilage rings were then filled with hydrogel solution **(F)** and secured in the bioreactor by a PDMS-holder and fine needle **(G)** to undergo cyclic compression in an Ebers TC-3F bioreactor **(H)**.

Condyles were also received from consenting patients undergoing total knee replacement surgery with grade 3 osteoarthritis according to the Kellgren-Lawrence grading scale [HREC/16/SVHM/186]. Note that the condyles and hADSCs were received from different patients. To prepare cartilage discs, an 8 mm biopsy punch (Acu-Punch, Acuderm, Fort Lauderdale, FL, United States) was used to score cartilage down to the subchondral bone. Discs of cartilage were then removed from the bone using a scalpel (Figures 1A–D), rinsed in sterile PBS, and then incubated in a 12 well plate (Cellstar, Greiner Bio-One, Kremsmünster, Austria) with 3 mL/well of medium prior to commencing the experiment (DMEM/F12 (1:1) (Gibco) containing pen-strep and 10 μg/mL fungin [InvivoGen, San Diego, CA, United States)]. To prepare cartilage rings for the *ex vivo* model, a 4 mm biopsy punch (Acu-Punch, Acuderm) was used to create a chondral “defect” in each disc of cartilage the day prior to casting the cell-laden material (Figures 1A, E).

### 2.4 Preparation of cartilage-scaffold constructs

Sterilised GelMA was reconstituted in PBS containing pen-strep to a stock concentration of 12.5% w/v. Aliquots were prepared to achieve final GelMA concentrations of 7 and 8% w/v with the remaining components. LAP was added to a final concentration of 0.05% w/v, microbial transglutaminase to a final concentration of 1 U/mL (except for transglutaminase free controls), and hADSCs were included to a final concentration of 5 million cells/mL. For this study, compositions of 8% GelMA and 7% GelMA with transglutaminase (denoted as 7% GelMA + TG) were chosen to control for the stiffness and degradation properties of the material and assess the effect of transglutaminase in terms of improved adhesion compared to a transglutaminase free control (Trengove et al., 2021). Cartilage rings were filled with 30–40 µL of cell-laden GelMA solution (depending on the thickness of tissue) and irradiated for 30 s in a UV crosslinking box (BioLambda, São Paulo, Brazil) at an intensity of 20 mW/cm^2^ and wavelength of 405 nm (Figures 1A, F). Once crosslinked, samples were placed in a 12 well plate containing PBS to rinse for 5–10 min and complete filling of the defect was visually verified by brightfield microscope. PBS was then replaced with growth media and samples were placed in a 37°C/5% CO2 incubator overnight, allowing the hydrogel to swell and dissolution of any uncrosslinked material. The experiment was repeated twice with an equal number of samples of each hydrogel condition (8% GelMA or 7% GelMA + TG) and each culture condition (free swelling or loaded) tested across the two experiments combined (n = 6 per condition). Cellular scaffold only controls were formed in polydimethylsiloxane (PDMS, Sylgard 184, Dow, Midland MI, United States) molds of comparable dimensions to the model cartilage defects for each experiment and kept in static culture conditions (free swelling).

### 2.5 Dynamically loaded chondrogenic culture of cartilage-scaffold constructs

A three chambered bioreactor (EBERS TC-3F, EBERS Medical Technology, Zaragoza, Spain) designed to apply displacement-controlled uniaxial cyclic compression was used, with two samples per chamber. Anchored, PDMS rings were designed in Solidworks (Dassault Systèmes, France) and fabricated to secure cartilage-scaffold constructs during culture in the bioreactor (Figure 1G). These were fabricated using a previously published negative sacrificial template (NEST3D) method (Doyle et al., 2021). PDMS rings were looped under metal platforms in the bioreactor chamber upon which samples were placed, and a 30 gauge sterile needle (Novofine, Novo Nordisk, North Sydney, NSW, Australia) was then used to pierce both the PDMS ring and cartilage to secure the construct in place. Assembly of bioreactor chambers was completed according to manufacturer’s instructions. A 0.2 μm pore size hydrophobic PTFE gas exchange filter (Minisart, Sartorius Stedim, Gottingen, Germany) was attached to the top port, and a three way tap (BD ConnectaTM, BD, Helsingborg, Sweden) and 33 mm diameter polyethersulfone syringe filter with 0.22 μm pore size (Millex-GP, Merck, Darmstadt, Germany) to the bottom port, which was capped. The chamber was carefully attached to the bioreactor frame and filled with 20 mL chondrogenic media (Figure 1H). Chondrogenic medium consisted of high glucose DMEM (4,500 mg/mL, Sigma Aldrich, Castle Hill, NSW), pen-strep, fungin, 1X GlutaMAX (Gibco), 15 mM HEPES, 1% insulin-transferrin-selenium (Sigma Aldrich), 100 nM dexamethasone (Sigma Aldrich), 50 mg/mL ascorbic acid-2-phosphate (Sigma Aldrich), 10 ng/mL transforming growth factor-β3 (TGF- β3) (Peprotech, Rocky Hill, NJ, United States), and 10 ng/mL bone morphogenetic protein 6 (BMP6) (R&D Systems, Minneapolis, MN, United States). Once all three chambers were attached to the bioreactor, the pistons were lowered to contact the samples, and the vertical position of the lateral chambers was adjusted until a similar baseline force was registered by the three separate load cells (one per chamber). Static samples were placed in spare bioreactor chambers that were not attached to the bioreactor, allowing samples to float freely in the same volume of media. These free swelling samples were also pierced with a fine needle attached to a PDMS ring.

Samples were cultured for 28 days. Half the media within each chamber was changed every four to 6 days, with the used media stored at −80°C for later analysis. For samples under cyclic compression, the entire construct (the cell-laden hydrogel and cartilage ring combined) underwent loading for 1 hour a day, 6 days a week. First, a pre-strain of approximately 0.5%–0.6% (dependent on the thickness of each sample) was applied via a 0.01 mm displacement of the actuator. This was followed by 1 hour of cyclic compression at a frequency of 0.5 Hz and a 0.15 mm maximum displacement each cycle (approximately 5%–10% strain depending on the thickness of each individual piece, and ∼1 N load). The frequency and magnitude of compression were chosen based on literature data and are within a physiological range (Wong and Carter, 2003; Sanchez-Adams et al., 2014; Vainieri et al., 2018; Yodmuang et al., 2019). Between loading cycles, the pistons were raised at least 2 mm above the cartilage to allow the free flow of media over the samples.

### 2.6 Histology and immunohistochemistry

**FIGURE 2:**
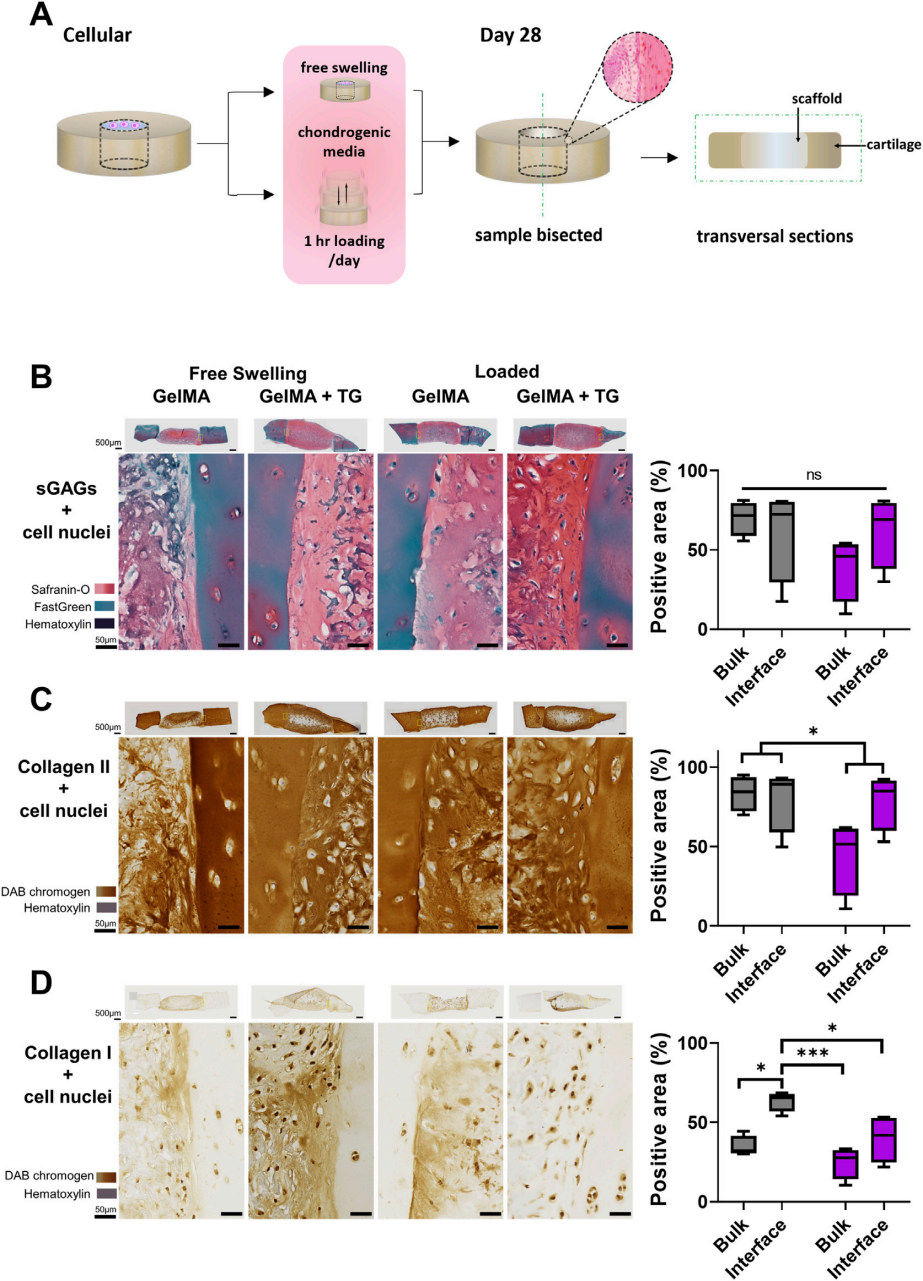
Histological and immunohistochemical staining of cartilage-scaffold cryosections. **(A)** Experimental workflow depicting orientation of cryosectioning. Representative brightfield images of stained cryosections and quantification of scaffold component from cartilage-scaffold constructs cultured in a bioreactor for 28 days. All free swelling and loading samples were analysed with ROIs that covered the full depth of the sample. Note the data is grouped by loading condition and location but not transglutaminase content, as ANOVA found that the absence or presence of transglutaminase was not a significant source of variation (Safranin-O: p = 0.79, type II collagen: p = 0.79, type I collagen: p = 0.32). **(B)** Safranin-O staining indicative of the presence of sGAGs. Immunohistochemical staining indicative of type II collagen **(C)** and type I collagen **(D)**. Box and whisker plots indicate the mean and interquartile ranges (n = 4). Significance assessed by ANOVA and Bonferroni post-hoc tests. Note that **(B)** shows the significant main effect of the loading condition, not post-hoc tests. ***p < 0.001, *p < 0.05.

After the 28 days culture period, representative samples were chosen for histology based on gross observations of the opacity of samples under a microscope. Samples were washed in PBS three times for 10 min each and fixed in 1% paraformaldehyde (Santa Cruz Biotechnology, Dallas, TX, United States) on gentle rocking at room temperature until the following day. Samples were once again rinsed in PBS, cut in half with a scalpel (creating two half-moon shaped pieces) and stored in 30% sucrose (Sigma-Aldrich) at 4°C for at least 1 week prior to embedding in O.C.T TM Compound (Tissue-Tek, Sakura, Leiden, Netherlands). Cryosectioning was performed by the Melbourne Histology Platform (University of Melbourne, Melbourne, Australia). Rectangular sections of 7 μm thickness were cut vertically, allowing the full depth of the lateral interface between the scaffold and cartilage to be visualized in each section (Figure 2A). Cryosections were mounted on SuperFrost glass slides (Trajan, Ringwood, VIC, Australia) for staining and imaging. Safranin-O/Fast Green/Hematoxylin staining was performed on all sections on the same day following the protocol described in Duchi et al. (2019). Cryosections were also stained on the same day for type I and II collagens using a Novolink Polymer Detection kit as per manufacturer’s instructions (Leica Biosystems, Wetzlar, Germany), for details refer to Supplementary Method 1.2. Stained sections were imaged with an Axioscan7 slide scanner (Zeiss, Oberkochen, Germany). The percentage of the section stained for Safranin-O, type II or type I collagen was then quantified using FIJI ImageJ software (NIH, US). A colour threshold was applied to quantify the number of pixels stained relative to the total number of pixels in the section. The threshold was applied in the CIELab colour space, as shifts within the colour space are proportional to the visually perceived difference in colours by humans (Kahu et al., 2019). The same threshold levels were applied to all images analysed. An example of this analysis is shown in Supplementary Figure S1. For each sample, this method was applied for six regions of interest (ROIs) that covered the full depth of the sample and were each 500 μm in width. Two ROIs covered the bulk of the cartilage, two ROIs the bulk of the scaffold, and two ROIs covered the interfaces between cartilage and scaffold. A 100 μm thickness was then manually selected to assess the area of scaffold immediately next to the interface.

### 2.7 Mechanical testing of integration

**FIGURE 3:**
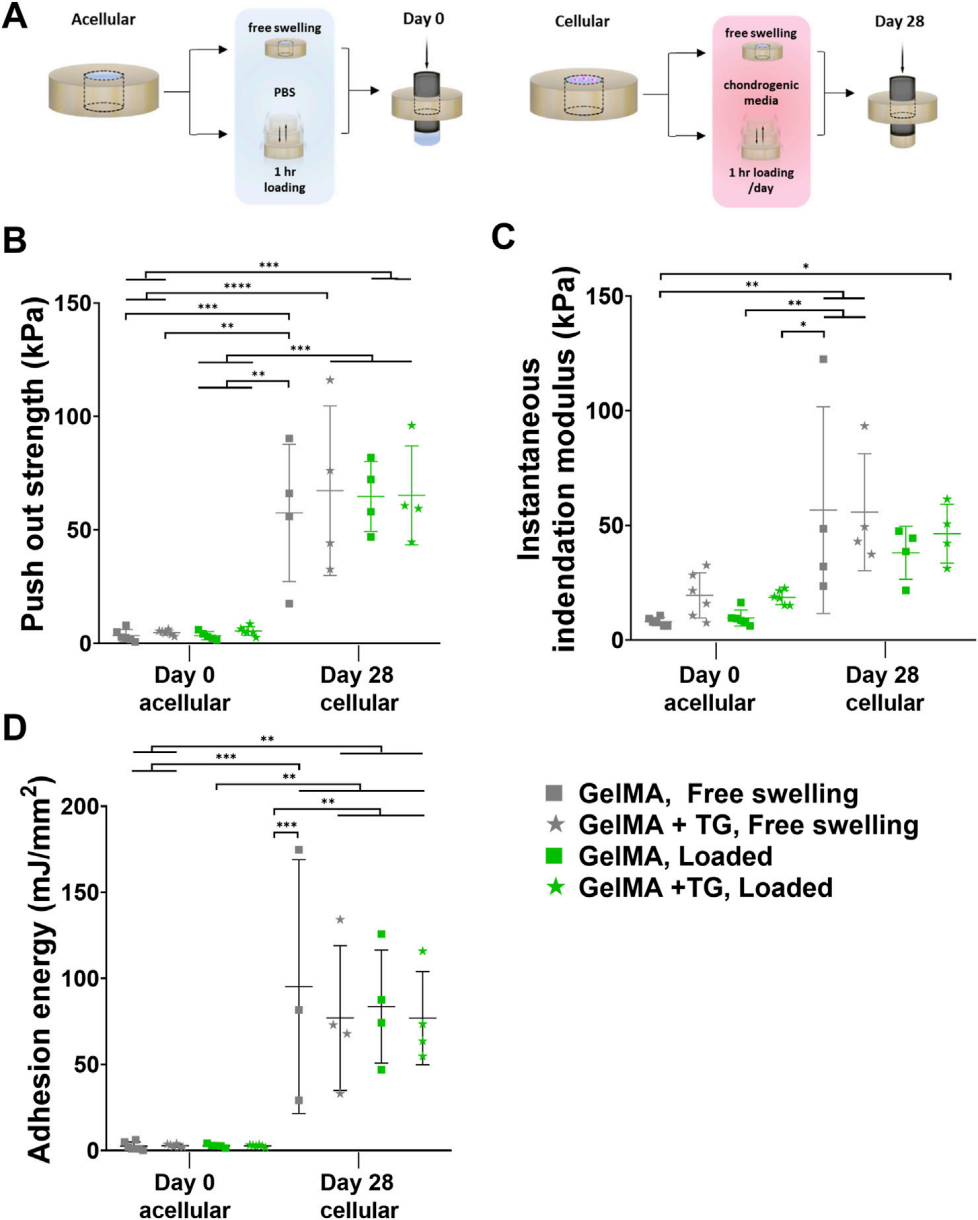
Mechanical testing of *ex vivo* samples cultured in bioreactor. Results for 8% GelMA and 7% GelMA + TG *ex vivo* constructs cultured under free swelling and loaded conditions for 28 days. **(A)** Experimental workflow for push out testing. **(B)** Push out strength (kPa) as a measure of integration strength and compared to acellular day 0 controls. Due to the limited availability of tissue, day 0 controls cannot be performed with the same patients’ tissue as that used for bioreactor culture. **(C)** Instantaneous indentation modulus (kPa). **(D)** Adhesion energy (mJ/mm^2^). Individual data points are plotted with mean and standard deviation (n = 3, 4 for day 28, and n = 5, 6 for day 0). Significance was assessed by ANOVA, with Bonferroni post-hoc tests. ****p < 0.0001, ***p < 0.001, **p < 0.01, *p < 0.05.

A TA ElectroForce 5,500 mechanical testing device (TA Instruments, New Castle, DE, United States) was set up for performing an indentation test (with a 250 g load cell) followed by a push out test (with a 220 N load cell). Samples were removed from the incubator to acclimatize to room temperature for a minimum of 30 min prior to testing. The indentation test was performed using a 1 mm diameter stainless steel cylindrical indenter to a strain depth of no greater than 10% (with variation due to the different thicknesses of the samples). This allowed for accurate calculation of the height of the scaffold within the cartilage ring by identifying the point of contact between the indenter and the top of the scaffold. This indentation was performed at a strain rate of 0.01 mm/s and a custom Python program was used to identify the inflection in the load-displacement curve indicating contact with the top of the scaffold. Instantaneous indentation modulus was calculated consistently over the 0%–5% strain range. After this test, samples were immediately returned to PBS to maintain hydration. The push out test was performed using a previously described protocol, with a 3.4 mm diameter indenter lowered at a strain rate of 0.01 mm/s to dislodge the scaffold from the cartilage defect (Figure 3A) (Trengove et al., 2021). The contact area between the gel and the inner surface of the cartilage defect was calculated as the circumference of the cartilage defect multiplied by the height of the scaffold. The maximum force to dislodge the sample was normalized by the contact area to calculate the push out strength. Adhesion energy was calculated as the area under the curve up to the failure displacement.

### 2.8 Media analyses

Sulfated glycosaminoglycans (sGAGs) were detected in the media via a dimethylmethylene blue (DMMB) assay. DMMB reagent was prepared by dissolving 3.2 mg DMMB (Sigma Aldrich), 0.6 g glycine (Sigma Aldrich) and 0.32 g sodium chloride (Sigma Aldrich) in 19 mL of 0.1 M acetic acid (Sigma Aldrich), and completing the volume to 200 mL with distilled water. Chondroitin sulfate (Sigma Aldrich) was dissolved in basal chondrogenic medium and used to prepare a standard curve from 0 to 1,000 ng. Standards and release media collected at each timepoint were thawed and combined with DMMB solution in a 1:5 ratio in a 96 well plate (Greiner, Sigma). The absorbance was immediately read using a CLARIOstar plate reader at 525 nm and 595 nm, and the ratio of the values at 525 and 595 nm was calculated. The total GAG amount per timepoint was calculated based on a standard curve generated with chondroitin sulphate (Sigma Aldrich).

Type II collagen, TGFβ3 and interleukin-6 (IL-6) in culture media were each quantified using enzyme linked immunosorbent assays (ELISA) (DuoSet, R&D Systems, Minneapolis, MN, United States) according to manufacturer’s instructions (for details refer to Supplementary Method 1.3).

### 2.9 Statistical analysis

Two trials of the bioreactor experiment were performed, with an equal number of samples of each hydrogel condition (8% GelMA or 7% GelMA + TG) and each culture condition (free swelling or loaded) tested across the two experiments combined (n = 6 per condition). One sample for each culture and hydrogel condition was reserved for histology per trial (sample size of n = 2 across both trials), and the remaining samples from each trial were used for mechanical testing (sample size of n = 4 across both trials). Statistical analyses were performed using Prism 8 (GraphPad Software Inc.). All values are reported as the mean and standard deviation, or where specified reported as median and interquartile range by box and whisker plots. Normal distribution of the datasets was assumed based on the Shapiro-Wilk test of normality (alpha = 0.05). Analysis of variance (ANOVA) was used to test for statistical significance between multiple groups (mechanical testing, media analyses and quantification of staining in scaffolds) with Bonferroni post-hoc tests, and a statistical significance level of 0.05. Unpaired t-tests were used to compare independent groups for quantification of staining in cartilage with a statistical significance level of 0.05.

## 3 Results

Cartilage ring-scaffold constructs were cultured in chondrogenic media for 28 days in the TC-3F bioreactor, with loaded samples undergoing daily cyclic compression, and free swelling samples that did not undergo mechanical stimulation serving as controls. After the 28 days culture period, the metabolic activity of cells within cartilage for both loaded and free swelling samples was comparable to that measured at day 0 (Supplementary Figure S2).

### 3.1 Accumulation of sGAGs and type II collagen

Safranin-O staining of cryosections was performed to identify sGAGs (counterstained with Fast Green and Hematoxylin). Immunohistochemical staining was performed to detect type II and type I collagen. Sections from each condition are shown in Figures 2B–D, representing the set of results from one trial of the experiment. The complete set of whole sections from both trials are found in Supplementary Figure S3. Quantification of the area stained in the bulk of the scaffold and at the interface with cartilage is also shown in Figures 2B–D.

Overall, the stained sections show evidence of the accumulation of sGAGs and type II collagen across each culture and gel condition. Areas indicative of sGAGs are stained positive for Safranin-O, appearing on a spectrum of intensity from light to dark pink, and areas of intense orange/red. Cell nuclei can be observed dotted throughout the sections, stained dark violet by Hematoxylin. There are areas of blue-green colour, particularly in the cartilage, due to Fast Green counterstaining for background proteins. Regions of dark blue/purple colour are indicative of stained undegraded GelMA.

A strong stain for type II collagen is seen in all samples relative to type I. There is almost no positive stain for type I collagen in the cartilage, whilst there is some type I collagen throughout the scaffold. However, the type II collagen stain is visibly stronger than type I and of a comparable intensity to the native cartilage. There are areas absent of type II collagen staining in similar location to areas of undegraded GelMA in the Safranin-O stained samples (dark blue/purple regions).

The gel condition (8% GelMA or 7% GelMA + TG) was not a significant source of variation for any of the three stains. No significant differences were observed in the positive area stained for Safranin-O. However, the areas stained positive for type II and type I collagen were reduced under the loaded condition as indicated by significant main effects (25% less staining for type II collagen, p-value = 0.0494 and 34% less staining for type I collagen, p-value = 0.006). 95% confidence intervals (CIs) and p-values for all post-hoc tests are shown in Supplementary Figure S4.

Images of the interface between cartilage and the scaffold reveal good contact with the cartilage rings. There is no evidence of gaps between newly generated ECM or residual scaffold and the cartilage explants. In the bulk region of the scaffolds there appears to be a consistent pattern of matrix accumulation at the top of the samples. This is observed for sGAGs and type II collagen, with accumulation of matrix less evident deeper into the samples. This is particularly apparent for some of the loaded samples, which were chosen to be thicker than free swelling controls to meet the requirements of the bioreactor (and with limited tissue available). The top edges of the samples also appear to have a denser distribution of cells than deeper regions.

### 3.2 Mechanical testing of *ex vivo* samples cultured in bioreactor

Results of mechanical tests of the *ex vivo* constructs are shown in Figures 3B–D. An indentation test was performed for accurate measurement of the thickness of each sample, also allowing for the calculation of an instantaneous indentation modulus up to 5% strain (Figure 3C). Based on one-way ANOVA, there were no significant differences between the loading nor gel conditions at 28 days. However, the modulus for each condition after 28 days was significantly higher than the GelMA, free swelling day 0 control, except for GelMA samples that underwent loading (but no addition of transglutaminase) (95% CIs of difference in means and p-values are given in Supplementary Figure S5).

Following the indentation test, a push out test was performed as a measure of the integration strength of each construct. These results were compared to acellular controls tested at day zero (Figure 3B). Samples cultured for 28 days saw an increase in push out strength of around 15-fold compared to day 0 controls (∼64 kPa at day 28 compared to ∼4 kPa at day 0). All day 28 conditions were significantly higher than day 0 results, with 95% CIs and p-values given in Supplementary Figure S6. For example, there was an almost 59.8 kPa increase in push out strength for loaded GelMA + TG samples after 28 days as compared to acellular day 0 controls (65.2 kPa ±21.8 kPa against 5.4 kPa ±2.0 kPa, p-value = 0.0002). No significant differences were observed between the loading or gel conditions at day 28.

Acellular scaffolds in cartilage rings were not cultured up to 28 days in this study, however previous data (Supplementary Figure S7) utilizing the same *ex vivo* model (human cartilage explants containing GelMA with or without hADSCs) cultured in a well plate saw no significant difference between the push out strength of acellular samples at day 0 and after 28 days chondrogenic culture (p-value > 0.999).

The adhesion energy was also calculated based on the area under the curve up to failure during the push out test (Figure 3D). Similar trends were observed as to the push out strength, where all day 28 conditions were significantly higher than day 0 results. After 28 days culture, mean adhesion energies increased from approximately 2.5–2.9 mJ/mm^2^ to 76–95 mJ/mm^2^ (refer to Supplementary Figure S8 for CIs and p-values).

### 3.3 Effect of chondrogenic culture on sGAG and type II collagen content of cartilage

**FIGURE 4:**
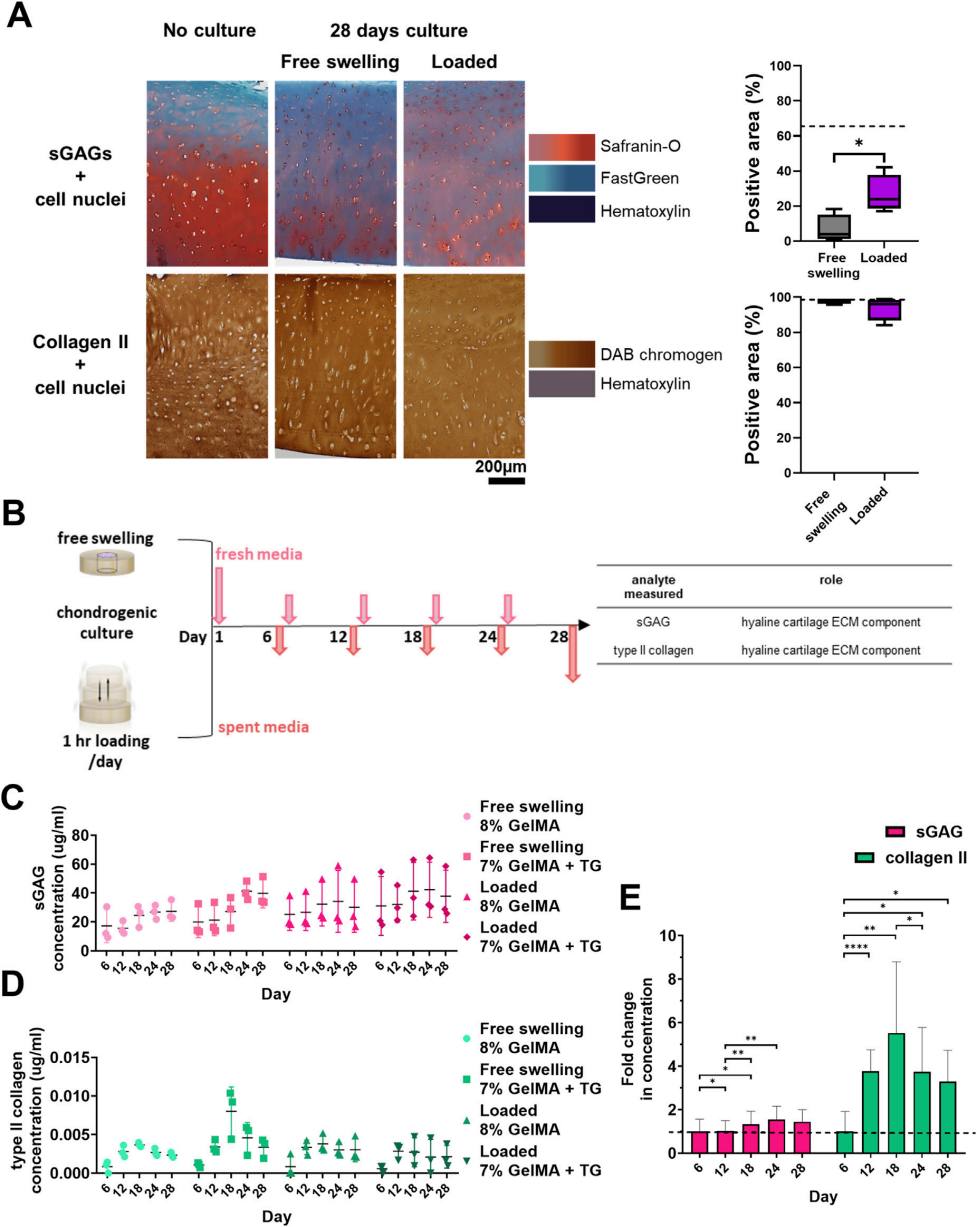
Cartilage maintenance and analysis of release media during bioreactor culture. **(A)** Safranin-O and type II collagen staining of cartilage and quantification of percentage of area stained positive for sGAGs and type II collagen. Dashed lines indicate the percentage area stained for no culture controls. Box and whisker plots indicate the mean and interquartile ranges (n = 4). Significance was assessed via student’s t-test, *p < 0.05. **(B)** Experimental workflow for collection of media during culture and analytes assessed. Concentrations of analytes in media during the 28 days culture period for each condition. **(C)** sGAG (µg/mL). **(D)** type II collagen (pg/mL). Individual data points are plotted with mean and standard deviation (n = 3). **(E)** Average relative fold change in concentration of each analyte over time for all the conditions analysed. Dashed line indicates one-fold. Mean and standard deviation are plotted (n = 12). Significance was assessed by ANOVA, with Bonferroni post-hoc tests. ****p < 0.0001, **p < 0.01, *p < 0.05.

Following staining of cryosections it was observed that native cartilage had areas lacking in sGAG, with a stronger than expected counterstain from Fast Green (Figure 4A). Cartilage tissue that had not undergone a period of chondrogenic culture was stained for comparison, showing intense Safranin-O staining relative to 28 days cultured samples. Vibrant areas of orange-red colour were observed emanating from chondrocytes within the tissue, particularly those in the middle and deep zones of the cartilage. Quantification revealed a significant increase in the percentage area stained for sGAGs for samples that underwent loading, from 6.8% ± 8.0%–26% ± 10.7% (p = 0.024). There were no clear qualitative or quantitative differences between the samples in terms of type II collagen staining compared to samples that had not undergone culture.

### 3.4 Detection of analytes in media

sGAG and type II collagen were evaluated as markers of neocartilage production via non-destructive media analyses (Figure 4B). In addition, TGFβ3 and IL-6 were monitored to detect changes in the mesenchymal associated secretome of the cells. Data is represented in Figures 4C–E; Supplementary Figure S9, showing the concentrations of analytes detected in the media over time. These molecules were at detectable levels at all time points despite the large volume of media (though there were some samples with zero detected for type II collagen in the early timepoints, and IL-6 at later timepoints). As media was retrieved for each chamber, the concentration detected represents the average of the two constructs within each chamber (and the combined concentrations resulting from both the native cartilage and the scaffold).

Based on ANOVA, the hydrogel condition (8% GelMA or 7% GelMA + TG) was not a significant cause of variability in any of the data. Time had a significant effect on sGAG (p = 0.003), type II collagen (p = 0.0007), and IL-6 concentrations (p = 0.0012) but not TGFβ3 concentration. The experimental trial (with different patient cartilage used for trials 1 and 2) did have a significant effect on both TGFβ3 (p < 0.0001) and IL-6 (p = 0.007) concentrations. Finally, the culture condition (free swelling or loaded) was only a significant cause of variability for TGFβ3 concentration (p = 0.005).

A comparison of the relative levels of the molecules over time was also made in Figure 4E; Supplementary Figure S9. The fold change in concentration relative to the average concentration at the first media collection (day six) was assessed via repeated measures (ANOVA). This analysis found that in addition to the effect of time, there was significant variability between the relative levels of molecules (p < 0.0001). For sGAG concentration, the fold change over day six at each timepoint remained steady and close to one, reaching a peak of 1.6 ± 0.6 on day 24. For type II collagen, the largest fold change was observed at day 18 (5.5 ± 3.3). The type II collagen concentration detected for all subsequent timepoints was at least three-fold that of the first timepoint. Post-hoc analyses of the effect of time within each group saw significant differences between the first two timepoints and days 18 and 24 for sGAGs. For type II collagen, all timepoints had a significantly higher fold change than day six, with a significant increase detected at day 12, and a significant drop from day 18 to day 24. TGFβ3 concentration remained steady over the culture duration, and IL-6 concentration declined over time to less than 20% of the initial concentration. CIs and p-values for all post-hoc tests are given in Supplementary Figures S10, S11.

## 4 Discussion

The aim of this work was to establish a dynamically loaded *ex vivo* model tailored for cyclic compression and mechanical analysis of the adhesion of a hydrogel implant to human cartilage where neocartilage formation is evident, and to investigate if microbial transglutaminase can support better integration under cyclic compressive load.

Stimulation of *ex vivo* models will impact the behaviour of chondrocytes within native cartilage, and likely the cells delivered to the defect for regeneration (Sah et al., 1989; Quinn et al., 1998; Gardner et al., 2016; Anderson and Johnstone, 2017; Klinder et al., 2020). Dynamic compressive loading may even support further integration if a pre-existing link exists between the scaffold and cartilage ring, as observed in a study where 28 days of static pre-culture was employed before cyclic loading of a construct (Yodmuang et al., 2019). Future work could investigate different loading conditions and their impacts on both integration and chondrogenesis in the *ex vivo* model, for which there is a gap in the literature, however such studies were not within the scope of this work. In this study loading provides a physiologically relevant environment (albeit simplified) by applying periods of cyclic compression to the construct. This is intended to act as a test of the interface, as may occur during continuous passive motion or partial weight bearing following surgery, though optimal post-operative care protocols for articular cartilage repair therapies are not yet fully defined (O'Connell et al., 2022). Strains applied in this study are comparable to physiological strains observed *in vivo* (Sanchez-Adams et al., 2014). The measured stress response (∼20 kPa) is much lower than that reported for studies of contact mechanics in the human knee (0.5–8 MPa) (Gilbert et al., 2014). This discrepancy likely highlights the limitations of the simplified loading regime (uniaxial, unconfined) in capturing the physiological environment of cartilage within the knee joint, which experiences multiaxial loading and time-dependent interstitial fluid pressurization (noting that comparison of stresses at different length scales may be difficult due to the complexity of scaling non-linear mechanical behaviours).

### 4.1 Histology demonstrated hyaline cartilage ECM components in the bulk and at the interface for all conditions

Histological and immunohistochemical staining demonstrated the production of sGAGs and type II collagen, the hallmark ECM components of hyaline cartilage, across all conditions (with or without transglutaminase, under free swelling or loaded culture). Minimal type I collagen was observed relative to type II indicating the hyaline rather than fibrous nature of the matrix. Based on histology, neocartilage was also well integrated with native cartilage.

Loading did not affect the positive area stained for sGAGs but did have a significant effect on the percentage area stained for both types of collagen, which were significantly lower under loading. Loading did not have a significant effect on the type II collagen/type I collagen ratio (Supplementary Figure S12), which was greater than one for all conditions, indicating the predominately hyaline nature of the neocartilage. It should also be noted that these quantitative results represent only two biological replicates for each condition, and thus these statistical differences may not hold practical significance. Overall, the use of microbial transglutaminase was not a significant cause of variability in any of the stains.

Based on gross observation these results were comparable to a study of human osteochondral explants cultured under static conditions for 28 days and stained for sGAGs and type II collagen (Kleuskens et al., 2022). A limitation of the data is that access to tissue was limited, and the thickest cartilage rings were reserved for culture in the dynamically loaded bioreactor chambers, to meet the minimum thickness requirement to use the system (∼ 1.3 mm). As a result, the free swelling samples tended to be thinner (and therefore filled with a smaller volume of cell-laden hydrogel). Matrix appeared to accumulate at the edge of scaffolds, potentially limited by diffusion of nutrients throughout the scaffold during chondrogenic culture (Heywood et al., 2006; Nims et al., 2014). Thinner samples may be less impacted by diffusion limitations than thicker samples, causing an underlying bias in the distribution of matrix throughout the scaffold. Whilst periods of loading should support the transport of nutrients into and out of the cartilage-scaffold constructs, these samples were also contained on one side by the metal platform rather than free floating in media. Regardless, neocartilage was produced for all conditions with no apparent differences due to the addition of microbial transglutaminase or loading based on this set of results.

Human cartilage tissue was sourced from consenting osteoarthritis affected patients undergoing joint replacement, however an ideal model to study cartilage repair therapies to treat traumatic defects would require tissue from younger patients with healthy cartilage. Regardless, neocartilage formation was evident even in the presence of this likely inflamed and arthritic tissue.

### 4.2 sGAGs in cartilage appeared depleted during culture based on histology

Based on histological staining sGAGs appear depleted in the native cartilage over the culture period (Figure 4A). A comparison with tissue that had not been cultured for 28 days illustrated the stark difference. “Fresh” cartilage explants exhibited a strong, deep red stain for Safranin-O with only the superficial zone lacking sGAGs, as is observed for other human cartilage explants in the literature (Kleuskens et al., 2022). The cultured samples on the other hand showed an overall lack of Safranin-O staining, with only patches of staining surrounding chondrocytes within the tissue.

This qualitative depletion of sGAG did not correlate with viability of the tissue, as there was no change in the metabolic activity of chondrocytes within the tissue over 28 days. Interestingly, quantitative image analysis of cartilage stained for Safranin-O indicated that the bulk region of loaded cartilage had a significantly greater percentage area stained for Safranin-O than free swelling samples. Physiologically relevant cyclic compressive loading is generally expected to have a positive impact on the production of sGAGs by chondrocytes (Anderson and Johnstone, 2017). The chondrogenic media the samples were cultured in may have also stimulated production of sGAGs, given sGAGs appeared to be emanating from chondrocytes in both free swelling and loaded samples.

The overall depletion of sGAGs in cartilage may indicate that the culture conditions are not optimal for maintaining the tissue in culture for such a long duration of time (approximately 5 weeks). There is no current consensus for the optimal culture media to maintain cartilage explants (Trengove et al., 2024). Despite the degradation of the osteoarthritic tissue and potentially inflammatory conditions, there was clear evidence of hADSC differentiation and neocartilage production.

### 4.3 Analytes in media were generally not impacted by loading or the use of microbial transglutaminase

The use of transglutaminase did not have a significant effect on the concentrations of neocartilage markers, sGAG and type II collagen. These markers were successfully detected from the media using the established parameters proposed for this dynamic *ex vivo* model by the relevant assays despite the large volume of culture media necessitated by the bioreactor (20 mL per chamber). sGAG concentrations hovered around a similar baseline throughout the experiment, with some significant relative differences between timepoints. Alternatively, type II collagen concentrations increased significantly after the first timepoint (over 3-fold from day 6 to day 12) and maintained this relative difference throughout the experiment. These concentrations represent sGAG and type II collagen from both the native cartilage and the scaffolds. sGAG concentrations may be a combination of sGAGs produced and secreted by the chondrocytes within native cartilage and hADSCs within scaffolds, and sGAGs released during the breakdown of the cartilage during culture (as observed by the depletion of sGAG in histology). In a previous experiment utilizing the same *ex vivo* model to compare acellular to cellular scaffolds in cartilage rings over 28 days, no significant differences in the sGAG concentration were observed between the conditions (Supplementary Figure S13A). This could explain why there was little change in sGAG concentration in the media over the baseline (day 6) value in Figure 4E – sGAG from native cartilage may have dwarfed the amount produced within the scaffolds. On the other hand, mature chondrocytes found in native cartilage are not expected to produce type II collagen at a fast rate, with the collagen network of cartilage considered a “permanent structure” even in osteoarthritic tissue (Heinemeier et al., 2016). In this sense, relative increases in type II collagen detected in the media may be largely due to newly produced matrix by differentiating hADSCs within the scaffolds. Results of the previous experiment support this, with type II collagen concentration in media significantly higher for cellular over acellular samples after 25 days chondrogenic culture (Supplementary Figure S13B), suggesting minimal type II collagen is released by the native cartilage itself.

The levels of sGAG quantified here were an order of magnitude higher than that observed in a study which cultured human cartilage explants (ranging from 3 to 5 μg/mL) (Klinder et al., 2020). Levels of type II collagen however were one to two orders of magnitude lower than detected in the same study (ranging from 100 to 500 ng/mL). The study does not report the volume of culture media used however, making it difficult to make a detailed comparison with the results reported. However, with the experimental conditions (media volume, ratio relative to scaffold size) we showed that quantitative analysis of neocartilage markers is feasible.

The analysis of the growth factor TGFβ3 and the cytokine IL-6 demonstrates the feasibility of analysing the cellular secretome in our experimental conditions. Successful quantification of both was possible over 28 days by media from the dynamic ex-vivo model. The experimental trial had a significant effect on levels of both TGFβ3 and IL-6, suggesting variability between the patient tissue used for each trial. Inter-patient variability has been observed in other studies maintaining human cartilage explants in culture (Dolzani et al., 2019; Kleuskens et al., 2022). Ideally, sample number would be increased to manage this variability, however this bioreactor model is limited to the dynamic culture of six samples at a time (two per chamber). A previous study also observed intra-patient location dependent differences, finding that cyclic compression of human osteoarthritic cartilage downregulated inflammatory markers IL-6 and IL-8 in tissue from some areas of the joint (Assirelli et al., 2022). Though loading did not have the same effect in this work, the decrease in IL-6 levels over time for all conditions could indicate a possible anti-inflammatory effect due to the chondrogenic culture media or presence of hADSCs. Further work is required to distinguish between IL-6 produced by chondrocytes within the cartilage rings and produced by the hADSCs. However, MSCs have previously been observed to have immunomodulatory effects on osteoarthritic cartilage (Sahu et al., 2021).

TGFβ3 levels were monitored throughout the culture period. In our study, loading had a significant effect on TGFβ3 concentrations in media, with average concentrations higher in the free swelling than loaded condition. Monitoring of TGFβ3 levels in the culture media could provide insights into the growth factor cellular uptake by the cells in scaffolds (Caballero Aguilar et al., 2024). TGFβ3 is expected to be under continuous depletion as cells become chondrogenic, and in turn the detected levels would remain relatively constant (Caballero Aguilar et al., 2024). In agreement to this, the TGFβ3 levels in our work remained virtually constant throughout the monitored time points. Differences in TGFβ3 concentrations could be due to a difference in cellular uptake (i.e., lower concentration levels in loaded samples may be due to a higher uptake of TGFβ3). Conversely, multiaxial loading was previously shown to induce activation of endogenous TGFβ1 secreted by MSCs encapsulated in tyramine-modified hyaluronic acid hydrogels (Behrendt et al., 2020). Addition of dynamic compression to TGFβ3 induced chondrogenesis has been seen to improve mechanical properties of MSC-laden agarose, however only when loading is applied after an initial 3 week pre-culture period (Huang et al., 2010). As discussed earlier, mechanical stimulation conditions were not optimized to stimulate chondrogenesis in our work. Bioreactors designed to simultaneously deliver compression and shear, and able to increase sample size per experiment would assist further investigations into loading conditions to evaluate integration (Meinert et al., 2017).

### 4.4 Mechanical testing revealed a significant increase in integration strength over culture period

For all samples there was qualitatively good integration and contact between neocartilage and native tissue observed via histology. Push out tests were also performed to quantify the integration, with significant increases in the push out strength over 28 days, compared to acellular day 0 controls. On average, push out strengths increased 15-fold after 28 days chondrogenic culture.

Microbial transglutaminase was previously observed to have a significant impact on adhesion to human cartilage *ex vivo*, increasing push out strength two-fold (Trengove et al., 2021). In the current study, microbial transglutaminase did not provide an advantage in terms of the push out strength for either culture condition. The initial adhesion that the enzyme provides was intended to stabilise the implant to mitigate any destructive effects of loading, or even to provide sufficient interfacial strength such that integration could improve under loading (Yodmuang et al., 2019). The 95% confidence interval for the difference in mean push out strength between 8% GelMA and 7% GelMA + TG under loading (−42.0 to 41.0 kPa) indicates the groups are not statistically significantly different and it is unclear if microbial transglutaminase had an effect. As the confidence interval is wide, it suggests the sample size may have been small giving the study limited power, necessitating larger sample sizes in future studies. A 15-fold increase in push out strength was observed over 28 days, suggesting the effect of chondrogenic culture may have overshadowed the previously reported initial benefit of microbial transglutaminase. These results highlight the power of the *in situ* biofabrication approach, casting a solution allows for complete filling of the defect, whereas implantation of a pre-fabricated or mature scaffold may not. This is important for integration as viable cells are able to generate new matrix directly at the interface with native cartilage, as was observed in this work.

The push out strengths reported in this work after 28 days culture in the bioreactor (mean values ranging from 57 to 67 kPa) were a similar magnitude to that observed in the literature for *ex vivo* rings cultured under compressive loading. Iseki et al. reported that after 21 days, fibrin scaffolds containing connective tissue progenitor cells saw push out strengths of approximately 33 kPa, significantly higher than day 0 controls (Iseki et al., 2019). PVA scaffolds seeded with chondrocytes cultured for 42 days (28 days static, followed by 14 days dynamically loaded) were dislodged with push out strengths around 50 kPa (Yodmuang et al., 2019). Dynamic compression of a cartilage-ring model using bovine articular chondrocytes encapsulated in GelMA-glycol chitosan and seeded in a ring of bovine cartilage saw a significant increase in push out strength (60.8 kPa ± 12.0 kPa) after 28 days compared to free swelling (52.6 kPa ± 13.3 kPa) and day 0 (38.1 kPa ± 3.9 kPa) controls (Paul et al., 2023). The push out strengths of these engineered scaffolds are orders of magnitude lower than that reported of intact cartilage 8 MPa (van de Breevaart Bravenboer et al., 2004), however, the minimum clinically relevant push out strength which may support long-term integration and repair is unclear. There was also a large degree of variability in the data, which is similar to that observed in other push out test data in the literature (Galarraga et al., 2021).

The observed variability may be due to several sources. Beyond potential biological variability discussed in 4.3, differences in geometry between samples may have impacted chondrogenesis (diffusion of nutrients through samples of different thickness) and loading (differences in applied strain). Additionally, it is important to note a major limitation of the push out test, that it is impacted by the bulk mechanical properties of the material being dislodged, making comparison of results between samples or studies of differing modulus ill-advised (Dhert et al., 1992). Performing indentation to determine the modulus of the core can help analysis, as in this work. Ideally multiple indentations are performed to average results (accounting for the likely heterogeneity of the sample), however this was not possible in this study. Future work could investigate ways to increase the throughput of this bioreactor system to manage variability in the data. This could also allow for the investigation of preliminary timepoints (e.g., 7 days, 14 days), to provide more detailed information about how the push out strength changes with chondrogenic culture (and matrix accumulation) over time, and if the initial increase in push out strength observed due to TG at day 0 is observed at these preliminary timepoints.

In this work, samples containing transglutaminase had a lower concentration of GelMA (7% GelMA + TG) to match the compressive modulus of the 8% GelMA only samples (Trengove et al., 2021). These results illustrate that a lower mass of polymer was able to generate a similar level of mechanical stability and integration. This can have important implications for future development in the use of enzymatic crosslinking methods as a supplement or alternative to light-based photocrosslinking that requires the use of synthetic compounds with potentially cytotoxic effects.

## 5 Conclusion

A dynamically loaded *ex vivo* model of cartilage repair was established. This model was used to test the hypothesis that addition of microbial transglutaminase would support better integration of a cell-laden GelMA scaffold with cartilage explants under loading than controls, by providing mechanical stability due to its adhesive ability. Samples were maintained under chondrogenic culture for 28 days with daily compressive loading or left as free swelling controls. Good integration was observed for all conditions as was accumulation of cartilage ECM components (sGAGs and type II collagen) based on histological and immunohistochemical staining. The addition of microbial transglutaminase did not have a significant effect on the push out strength after 28 days, nor were there any differences in push out strength based on the loading condition. The push out strength did however significantly increase on average 15-fold over day 0. These results illustrate the chondrogenic potential of this cell-laden hydrogel and the challenge of biological variability when culturing explanted tissue. Overall, this *ex vivo* model holds promise as an alternative method to provide a thorough assessment of cartilage repair strategies prior to costly *in vivo* studies.

## Data Availability

The raw data supporting the conclusions of this article will be made available by the authors, without undue reservation.
