# Supplementary material for: A dynamically loaded ex vivo model to study neocartilage and integration in human cartilage repair
Source: Front Cell Dev Biol. 2024 Sep 30;12:1449015. doi: 10.3389/fcell.2024.1449015 (PMC11471648; doi:10.3389/fcell.2024.1449015)
Supplement: Supplementary file 1 [file Table1.DOCX]

Supplementary Material

# Supplementary Methods

## Metabolic activity assay of cartilage

Whilst creating the defects, the 4 mm diameter cartilage biopsy was transferred to a 24 well plate (Cellstar, Greiner Bio-One) containing 1 ml of a 1:5 ratio of CellTiter-Blue ® Reagent (Promega, Madison, WI, USA) and DMEM/F12. Biopsies were incubated in media and reagent for four hours to allow the reagent to diffuse throughout the tissue. After four hours, supernatant was collected, and 100 µl of supernatant was dispensed in triplicate in a 96 well plate (Greiner Bio-One). Fluorescence of the supernatant due to the conversion of resazurin to resorufin by metabolically active cells was measured using a CLARIOstar plate reader (BMG LABTECH, Freiburg, Germany) at 550–15 nm excitation and 600–20 nm emission, focal length 7.0 and gain 1000. Fluorescence results were normalised by the wet weight of the cartilage. The metabolic activity of the cartilage ring tissue was also assessed at the end of the 28 day chondrogenesis experiment. Following mechanical testing to dislodge the scaffold, biopsies were taken from the cartilage ring component and metabolic activity was evaluated via the same method.

## Immunohistochemistry

Sections were placed in a rack and first rehydrated by two changes in 100% ethanol for three minutes each, one change of 95% ethanol for one minute, one change of 80% ethanol for one minute, and then five minutes in distilled water. Borders were drawn around the sections of interest with a liquid blocker pen (Nandai, Japan). Sections were placed flat and covered with ready-to-use proteinase K (Dako/Agilent, Santa Clara, CA, US) for antigen retrieval and incubated for 30 minutes at 37 °C. After this incubation slides were placed back into a rack and washed in PBS for two changes of five minutes each. The following steps were then performed at room temperature unless otherwise stated. First, Peroxidase Block was applied to neutralize endogenous peroxidase for five minutes, followed by two washes in PBS for five minutes each. The slides were then covered with Protein Block for five minutes, followed by two washes in PBS for five minutes each. A solution of the primary antibody (anti-collagen II, DSHB II6B3, Developmental Study Hybridoma Bank, 57 μg/ml or anti-collagen type I, MAB3391 clone 5D8-G9, Millipore, 1mg/ml ) was then prepared at 100x dilution in 2% bovine serum albumin (Sigma). Sections were then covered in primary antibody solution and incubated at 4 °C overnight. Following this, slides were washed twice in PBS for five minutes each. The secondary antibody (Post Primary) was applied and slides were incubated for 30 minutes before two washes in PBS for five minutes each. Slides were then covered in Novolink Polymer for 30 minutes. Slides were then placed in PBS on an orbital shaker with gentle rocking for two washes of five minutes each. DAB Chromogen diluted in Novolink DAB Substrate Buffer was then applied to the slides which were left to develop (three minutes for type II collagen, and five minutes for type I collagen). At three minutes, slides were quickly transferred to distilled water for five minutes to stop development. Slides were then covered in Hematoxylin for five minutes, before a final wash in distilled water (for five minutes). Slides were dehydrated in one change of 80% ethanol for one minute, one change of 95% ethanol for one minute, two changes of 100% ethanol for three minutes each, and finally cleared in two changes of xylene for five minutes each. Coverslips were mounted on the slides with mounting medium (Pertex, Grale HDS, Ringwood, Australia) and left to set overnight before imaging.

## Enzyme Linked Immunosorbent Assays

Capture antibody specific to each analyte (Type II collagen, TGFβ3 and IL-6) was coated on a 96-well plate microplate and left to incubate at room temperature overnight. Following incubation, the microplate was washed three times with a wash buffer (Quantikine ELISA wash buffer, R&D Systems) and the plate was blocked with a 1% bovine serum albumin solution (BSA, R&D Systems). After 1 hour incubation at room temperature the plate was washed with wash buffer, and 100 μl of sample (the thawed collected media) was added. At the same time, a range of dilutions of the analyte (pro-collagen II, TGFB3 or IL6, R&D Systems) were prepared in the blocking solution (1% BSA) and added to the plate as a standard curve. After two hours incubation, the plate was washed again with wash buffer and the relevant detection antibody (R&D Systems) was added and left to incubate for a further two hours. After washing with wash buffer, streptavidin-horseradish peroxidase (HRP) (R&D Systems) was added and incubated for 20 minutes. After washing with a wash buffer, a 1:1 mixture of hydrogen peroxide and tetramethylbenzidine (R&D Systems) was added to the plate and incubated for 20 minutes at room temperature. After adding 2 N sulfuric acid stop solution to the plate, the optical density of each well was measured at two wavelengths immediately using a CLARIOstar plate reader, with readings at 540 nm subtracted from readings at 450 nm. A standard curve was constructed using the plate reader’s MARS software (BMG Labtech), generating a four parameter logistic curve fit. The standard curve was used to calculate the concentration of each analyte in terms of pg/ml for each sample.

# Supplementary Data


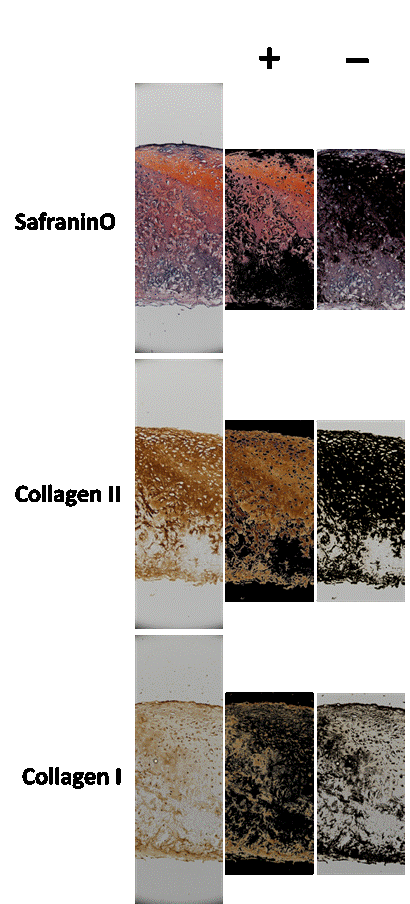


**Supplementary Figure 1.** Example of quantification of positive stained area using thresholds in the Lab colour space. Images show the segmentation of the image into the positively stained regions (+) and the remaining regions that did not stain for the relevant signal (-).


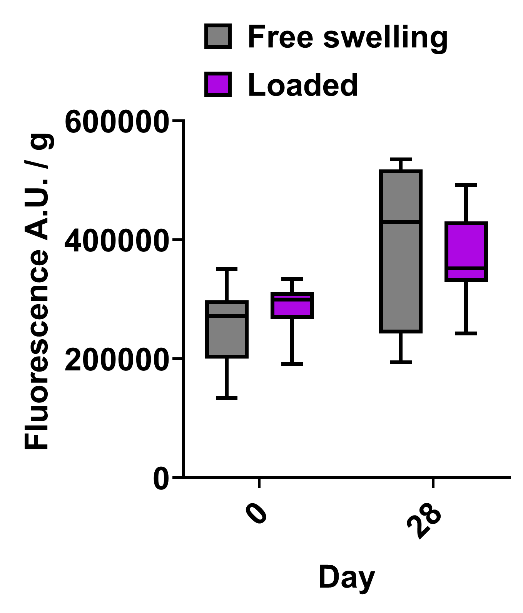


**Supplementary Figure 2.** Metabolic activity of cartilage explant before and after dynamic culture. Box and whisker plots show median and interquartile ranges (n=8).


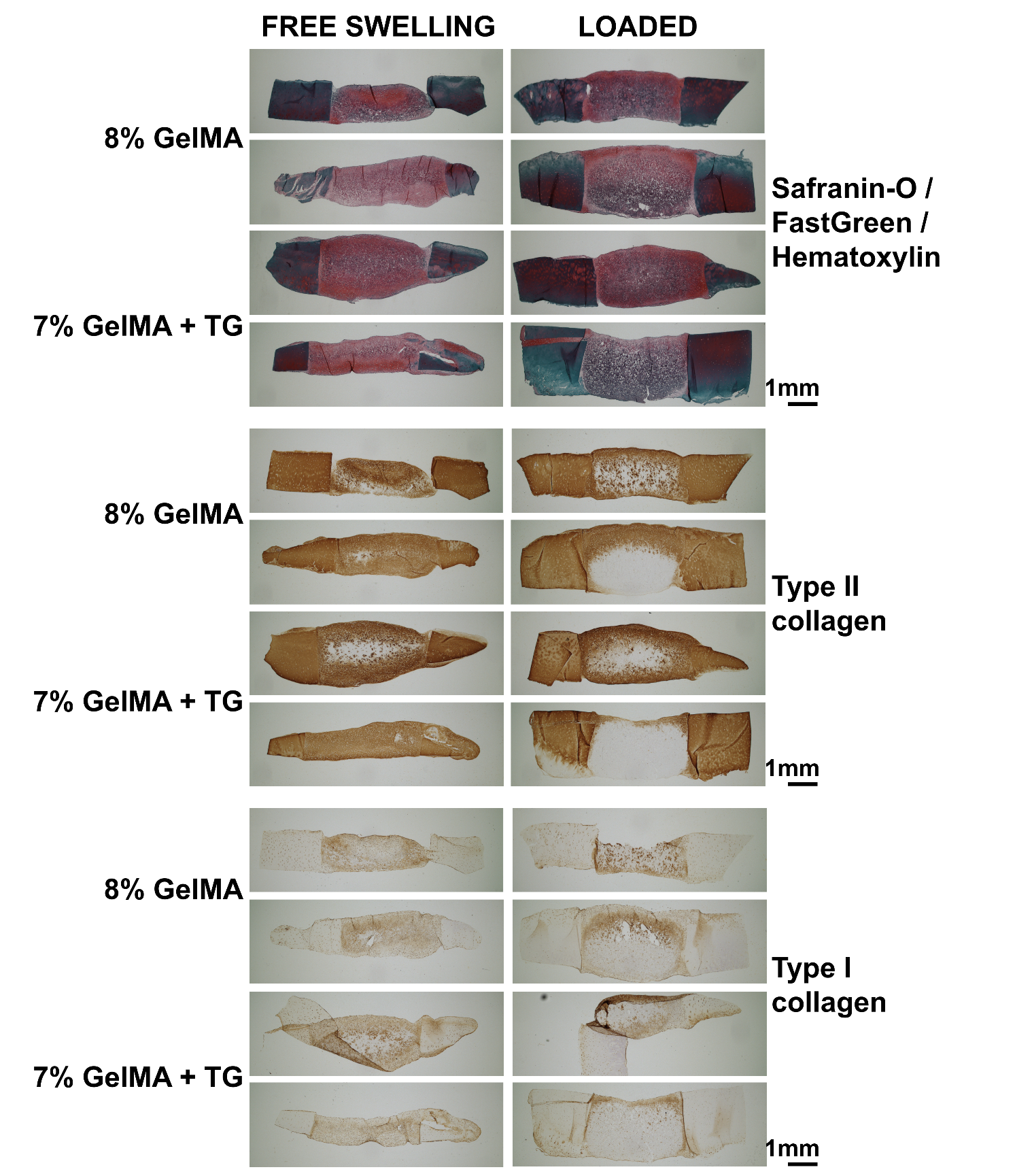


**Supplementary Figure 3.** Histological staining of the complete set of samples from the two bioreactor trials. Safranin-O/Fast Green/Hematoxylin (top) and immunohistochemical staining for type II collagen (middle) and type I collagen (bottom).


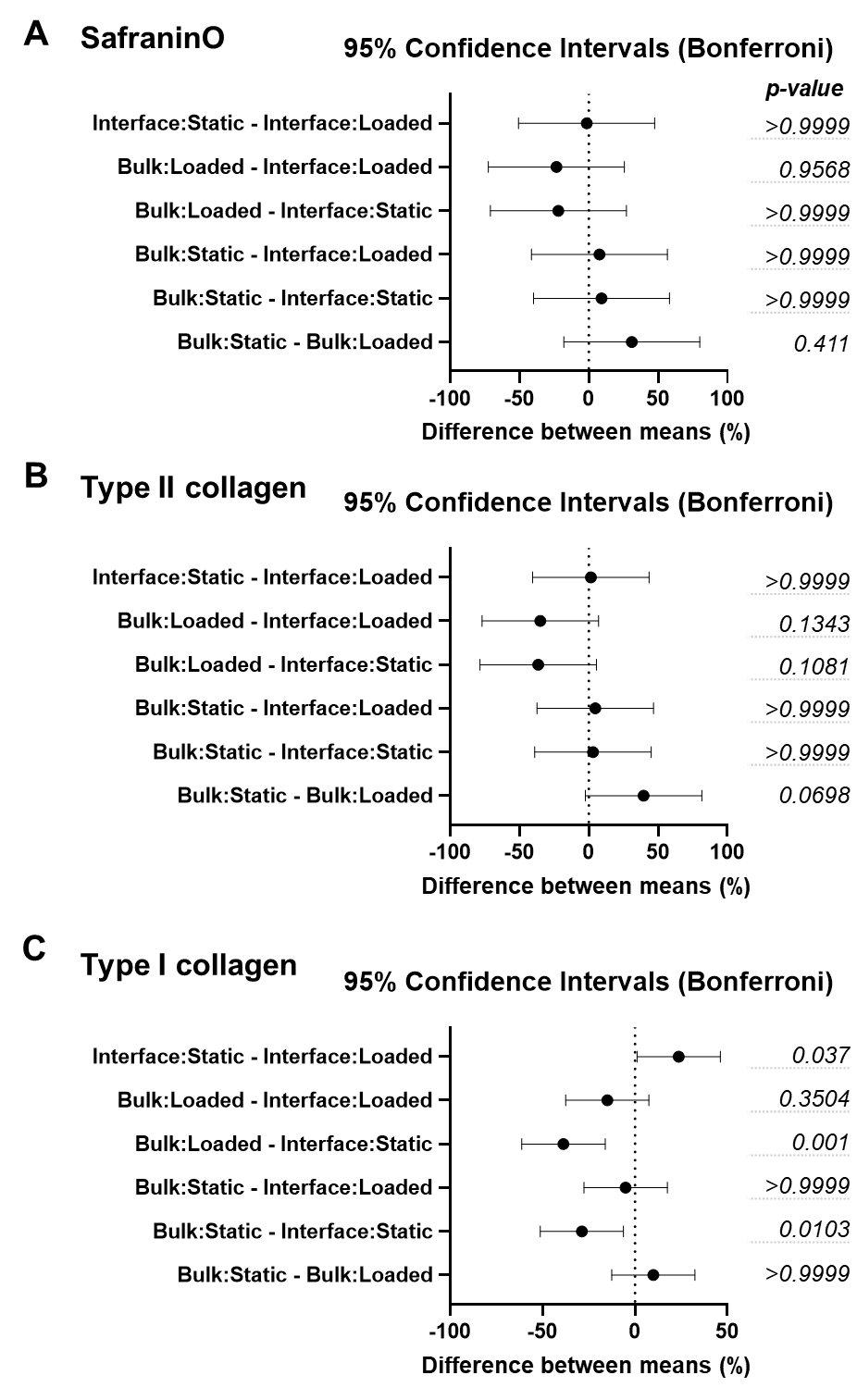


**Supplementary Figure 4.** 95% confidence intervals and p-values for all post-hoc tests of histology and immunohistochemistry image quantification data.


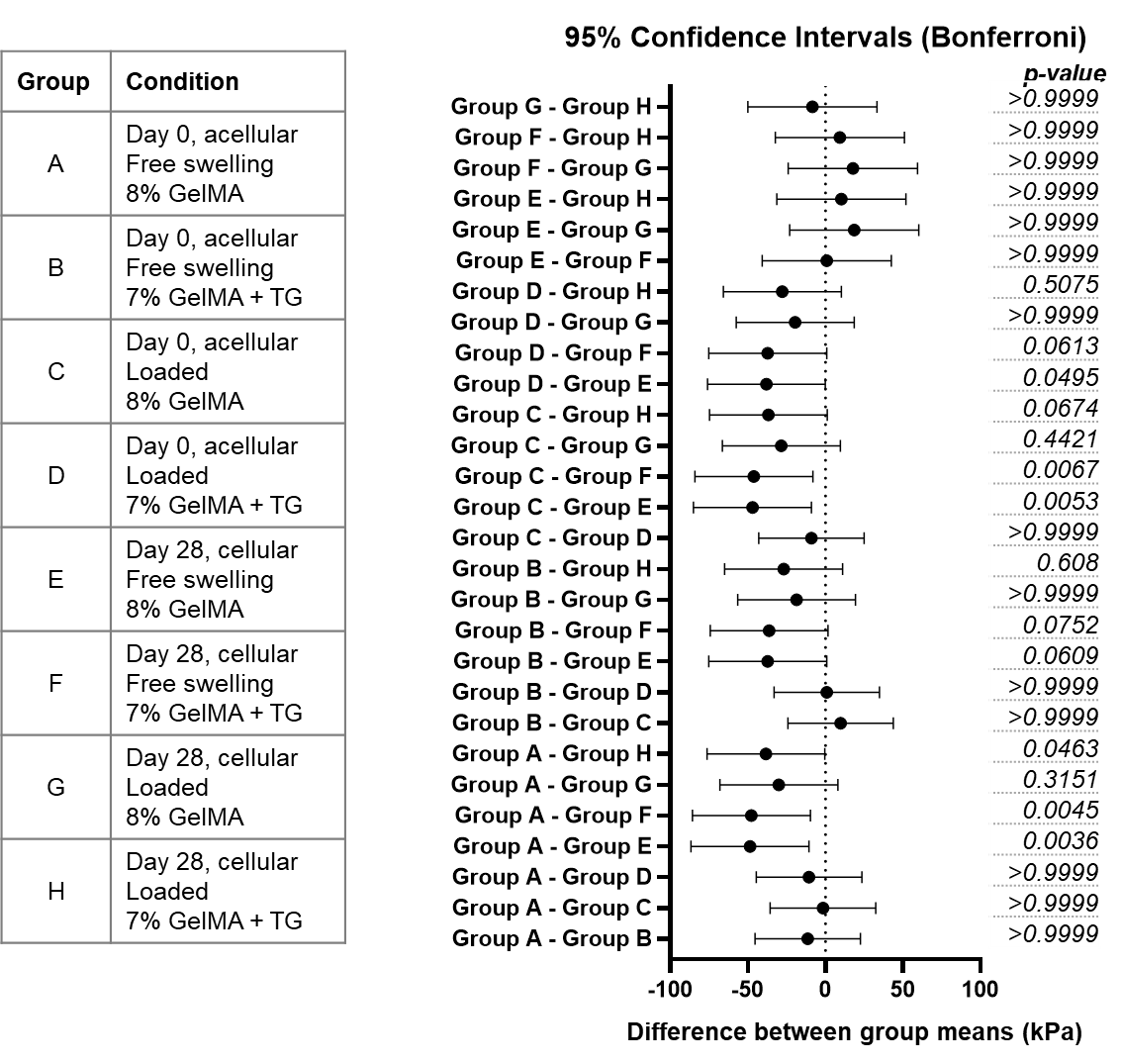


**Supplementary Figure 5.** 95% confidence intervals and p-values for post-hoc tests of instantaneous indentation modulus data.


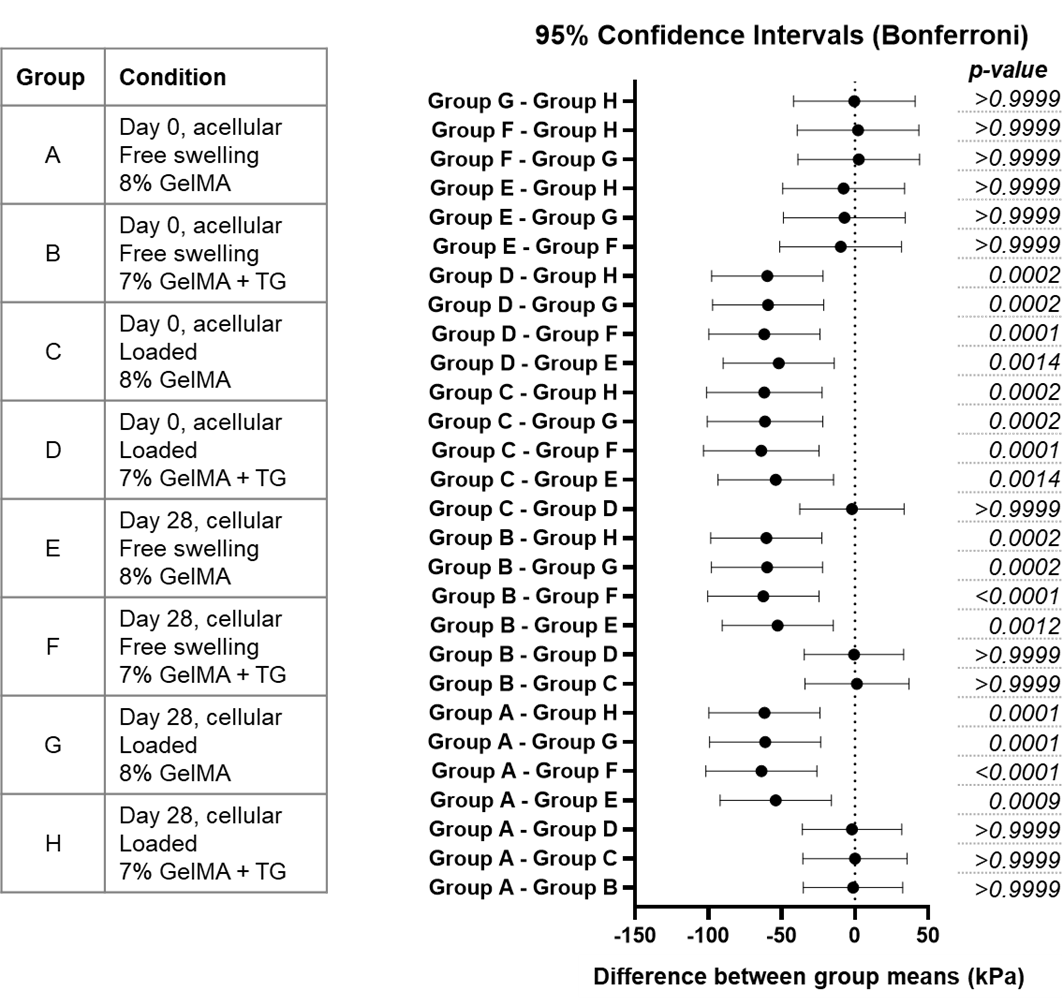


**Supplementary Figure 6.** 95% confidence intervals and p-values for post-hoc tests of push out strength data.


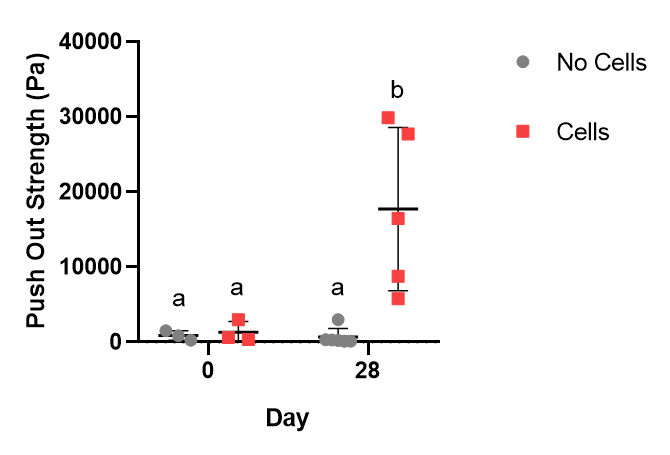


**Supplementary Figure 7.** Push out strength of ex vivo constructs cultured in a well plate comparing acellular and cellular scaffolds over 28 days. Individual data points are plotted with mean and standard deviation (n=3 for Day 0, n=5-6 for Day 28). Significance was assessed by ANOVA and Bonferroni post-hoc tests. For all conditions with the same letter, the difference between the means is not statistically significant.


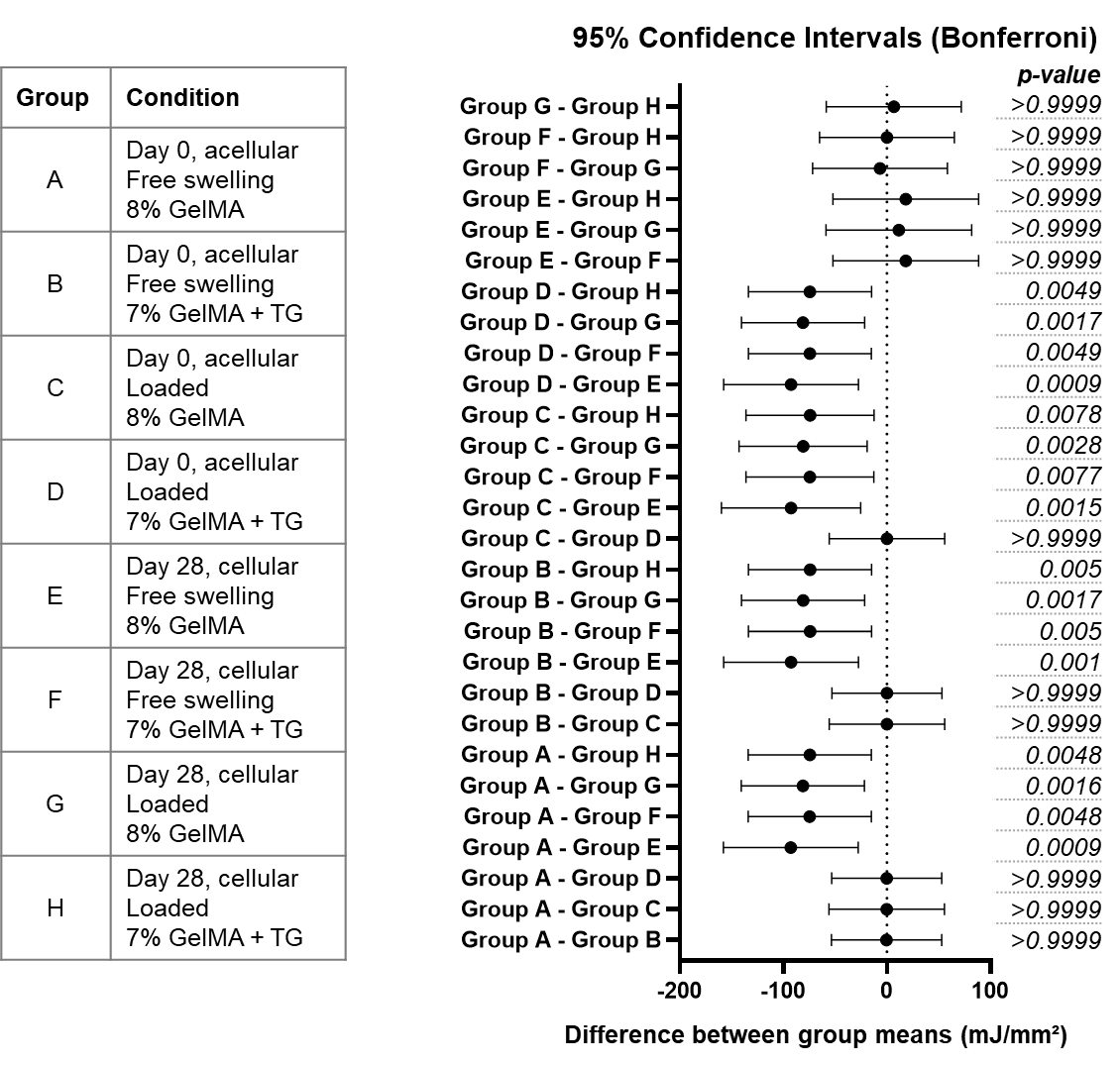


**Supplementary Figure 8.** 95% confidence intervals and p-values for post-hoc tests of adhesion energy data.


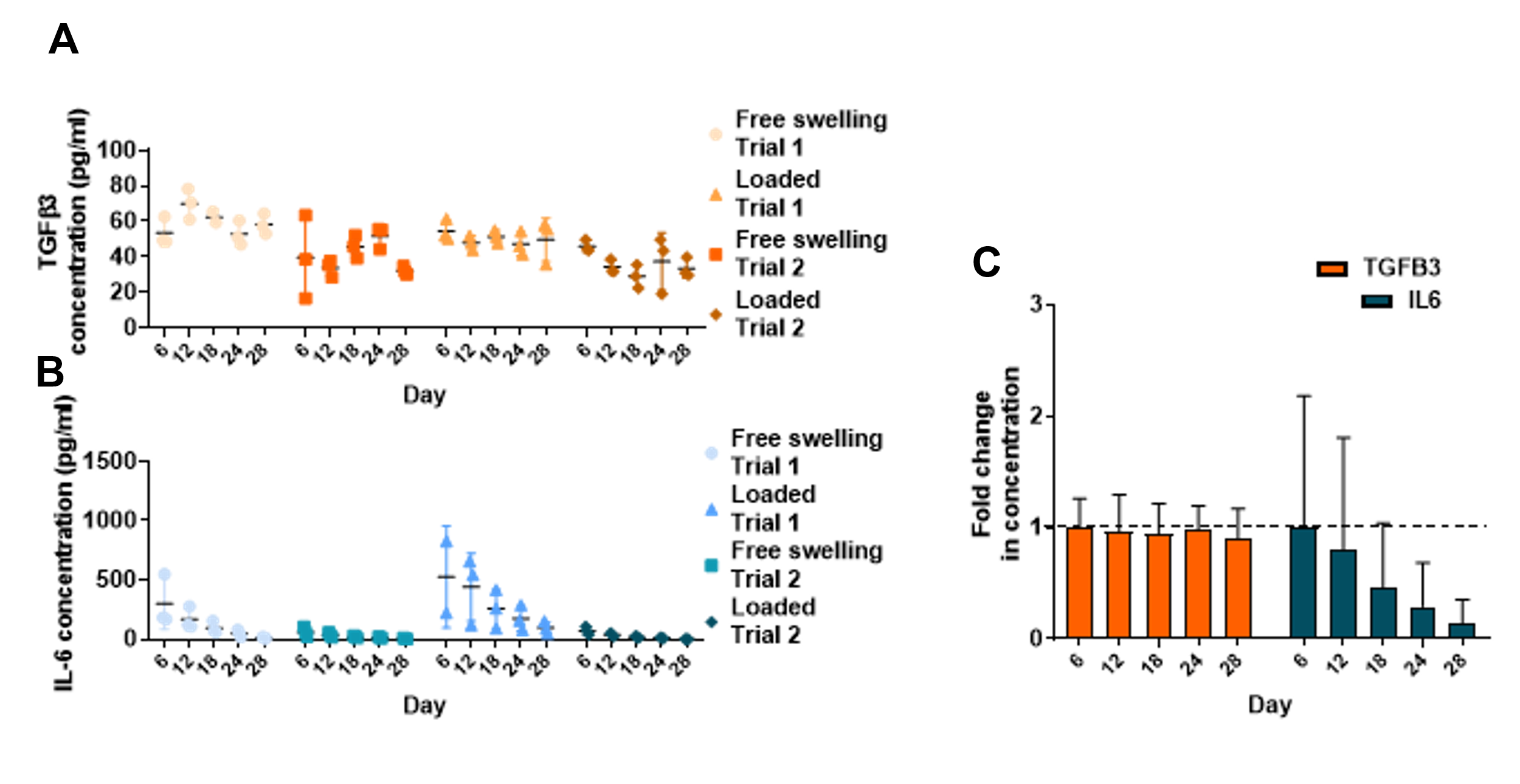


**Supplementary Figure 9.** Concentrations of analytes in media during the 28 day culture period for each condition. (A) TGFβ3 (pg/ml). (B) IL-6 (pg/ml). Individual data points are plotted with mean and standard deviation (n=3). (C) Average relative fold change in concentration of each analyte over time. Mean and standard deviation are plotted (n=12). Significance was assessed by ANOVA, with Bonferroni post-hoc tests.


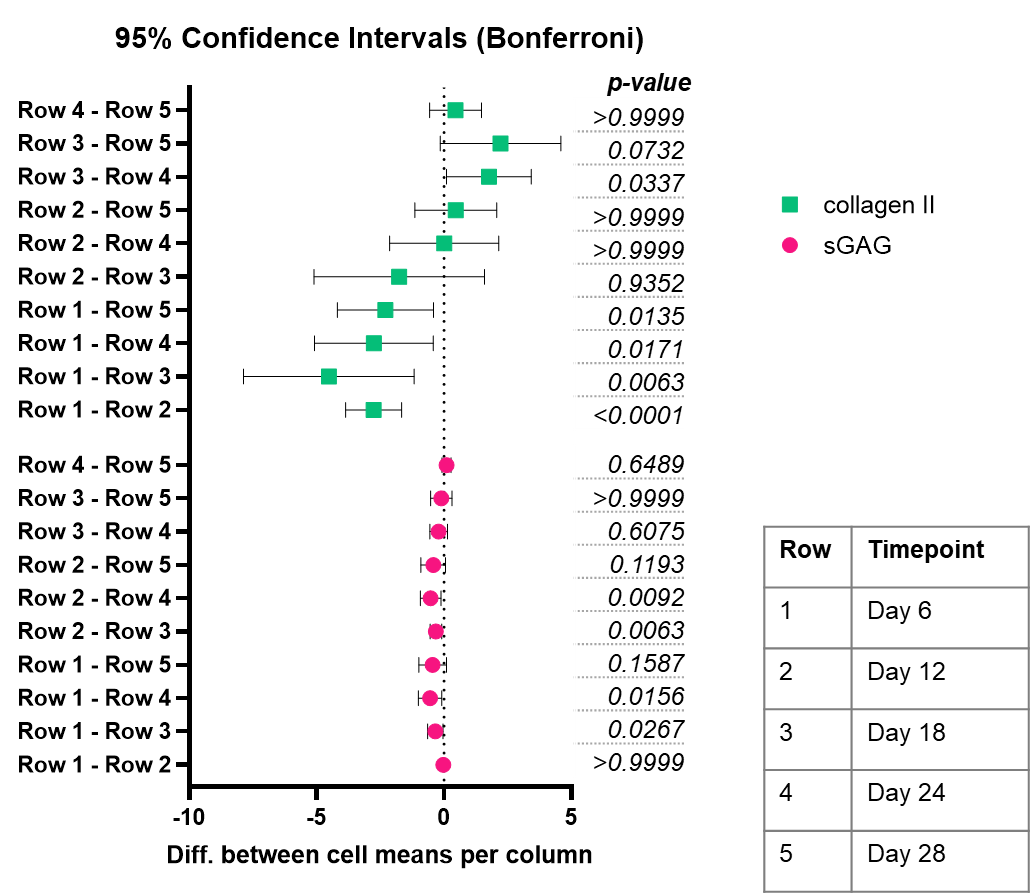


**Supplementary Figure 10.** 95% confidence intervals and p-values for post-hoc tests of relative levels of sGAGs and type II collagen detected in media over time.


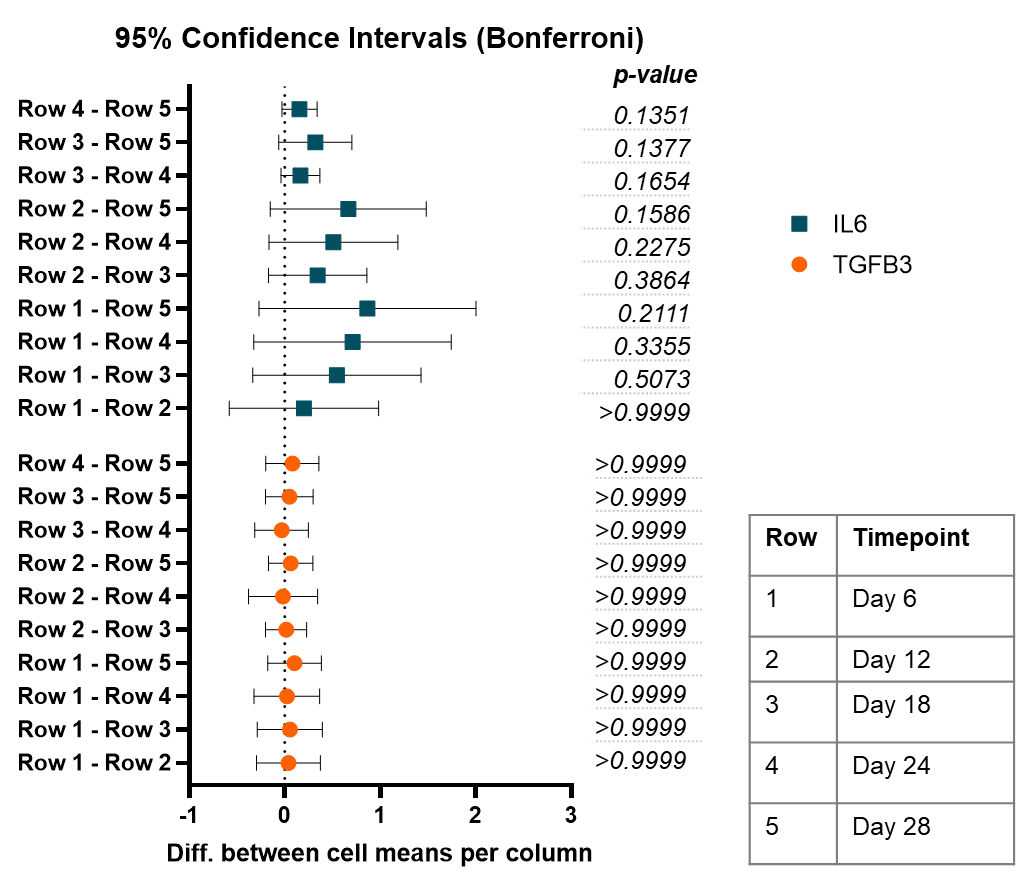


**Supplementary Figure 11.** 95% confidence intervals and p-values for post-hoc tests of relative levels of TGFβ3 and IL-6 detected in media over time.


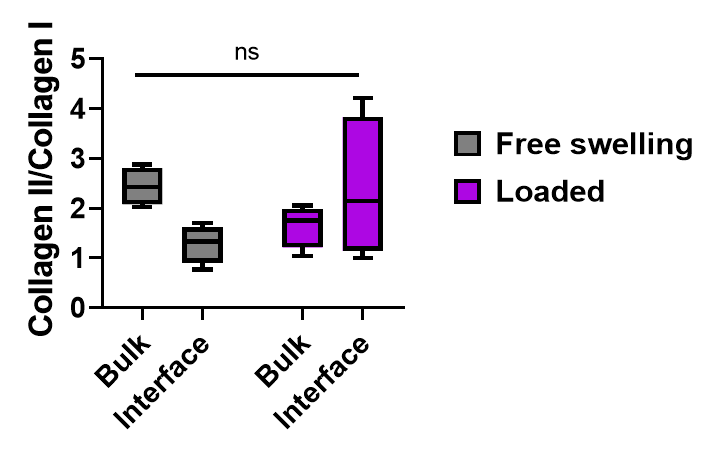


**Supplementary Figure 12.** Type II/Type I collagen ratio of scaffold component of stained sections from cartilage-scaffold constructs cultured in a bioreactor for 28 days. All free swelling and loaded samples were analysed with ROIs that covered the full depth of the sample. Box and whisker plots indicate the mean and interquartile ranges (n=4). Significance assessed by ANOVA and Bonferroni post-hoc tests.


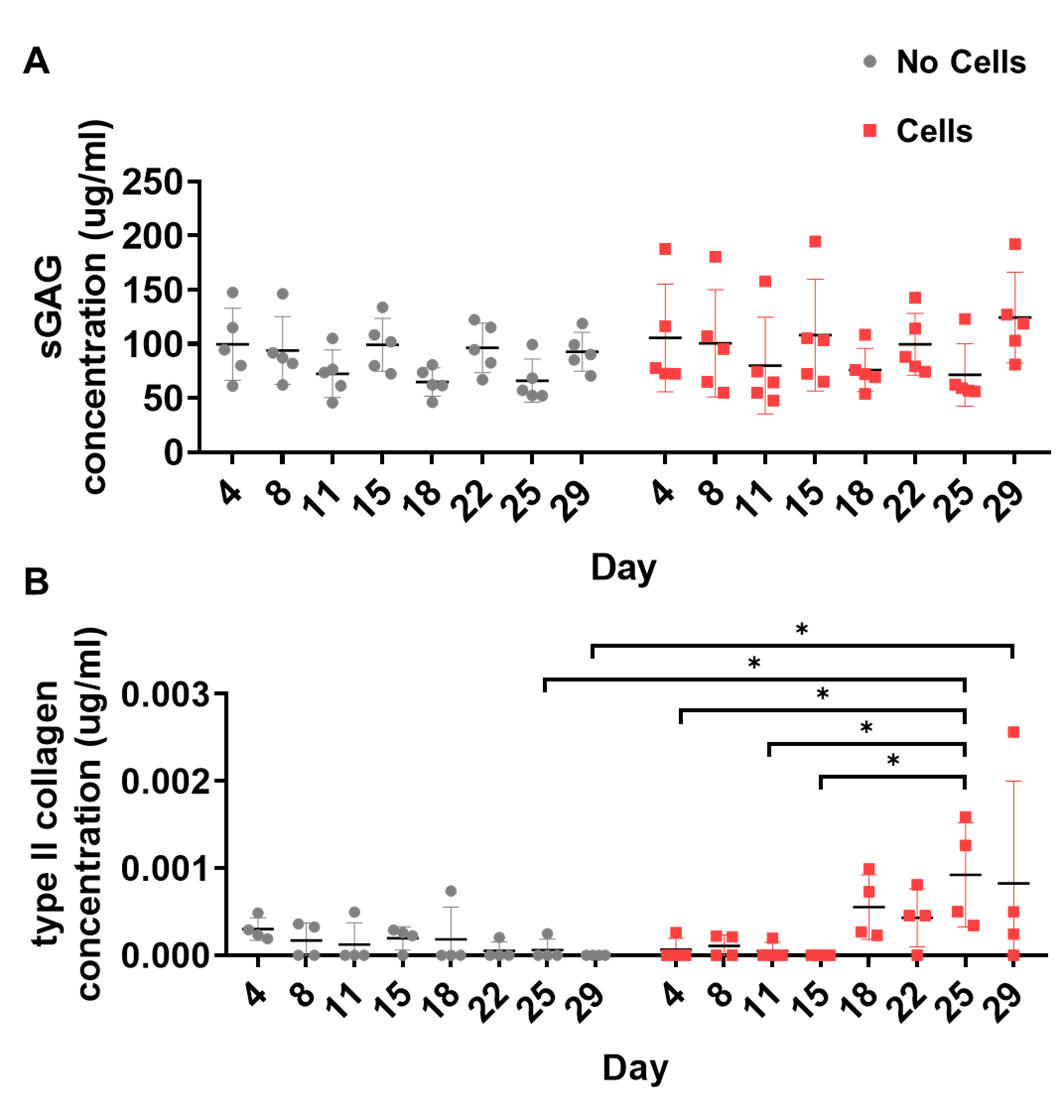


**Supplementary Figure 13.** Concentration of markers of neocartilage production for cartilage-ring constructs cultured in a well plate comparing acellular and cellular scaffolds over 28 days. (A) sGAG (µg/ml). (B) type II collagen (µg/ml). Individual data points are plotted with mean and standard deviation (n=4). Significance was assessed by ANOVA and Bonferroni post-hoc tests. *p<0.05.
